# Bioavailability of Coffee Chlorogenic Acids and Green Tea Flavan-3-ols

**DOI:** 10.3390/nu2080820

**Published:** 2010-07-29

**Authors:** Daniele Del Rio, Angelique Stalmach, Luca Calani, Alan Crozier

**Affiliations:** 1 Human Nutrition Unit, Department of Public Health, University of Parma, Via Volturno 39, 43100 Parma, Italy; Email: daniele.delrio@unipr.it (D.D.R.); luca.calani@nemo.unipr.it (L.C.); 2 Plant Products and Human Nutrition Group, Division of Developmental Medicine, Faculty of Medicine, Graham Kerr Building, University of Glasgow, Glasgow G12 8QQ, UK; Email: astalmach@hotmail.com

**Keywords:** coffee, green tea, chlorogenic acids, flavan-3-ols, human bioavailability

## Abstract

This paper reviews recent human studies on the bioavailability of chlorogenic acids in coffee and green tea flavan-3-ols in which the identification of metabolites, catabolites and parent compounds in plasma, urine and ileal fluid was based on mass spectrometric methodology. Both the chlorogenic acids and the flavan-3-ols are absorbed in the small intestine and appear in the circulatory system predominantly as glucuronide, sulfate and methylated metabolites. Even when absorption occurs in the small intestine, feeding studies with ileostomists reveal that substantial amounts of the parent compounds and some of their metabolites appear in ileal fluid indicating that in volunteers with a functioning colon these compounds will pass to the large intestine where they are subjected to the action of the colonic microflora. A diversity of colonic-derived catabolites are absorbed into the bloodstream and pass through the body prior to excretion in urine. There is growing evidence that these compounds, which were little investigated until recently, are produced in quantity in the colon and form a key part of the bioavailability equation of flavonoids and related compounds that occur in fruits, vegetables and beverages. Recent evidence indicates that some colon-derived phenolic acids have *in vitro* anti-inflammatory activity.

## 1. Introduction

Dietary phenolic compounds, comprise a diversity of flavonoids as well as simple and complex phenolic structures [1]. They are of substantial interest because of their perceived beneficial effects on health and are, arguably, among the most investigated group of compounds in nutritional research. 

Flavonoids and phenolic compounds undergo methylation, sulfation and glucuronidation after ingestion, with reactions that occur in the small and large intestine and in hepatic cells [1,2] Unmetabolised flavonoids appear to be absorbed only rarely with trace quantities of (–)-epicatechin-3-*O*-gallate and (–)-epigallocatechin-3-*O*-gallate being reported to appear in the circulatory system, but not urine, following consumption of green tea by humans [3]. Analysis of ileal fluid collected from ileostomists after the ingestion of various foodstuffs indicate that even when dietary flavonoids are absorbed in the small intestine, substantial quantities none-the-less pass from the small to the large intestine [4,5]. In the colon they are subjected to the action of the microflora resulting in cleavage of conjugating sugars and ring fission of the released aglycones followed by other metabolic steps giving rise to a series of phenolic acid catabolites that can be absorbed into the portal vein and excreted in urine in amounts corresponding to >20% of flavonoid intake [6,7]. Obtaining further information of the catabolism of polyphenols in the colon is emerging as a key target for research on the identity and origins of biologically active compounds derived from the diet. Microbial catabolites produced in this manner [8,9] may influence the local microflora, exerting prebiotic activity resulting in the selection of probiotic strains. In addition, the parent flavonoid molecules and the catabolites may be present in the colon in concentrations that have the potential to inhibit the proliferation of cancerous cells.

The aim of this article is to review more recent research on the bioavailability of dietary chlorogenic acids and flavan-3-ols, discussing the fate of these compounds as they pass through the body, being metabolised and absorbed in both the small and large intestine prior to urinary excretion. This will be done by reference to studies with coffee, which contains high concentrations of chlorogenic acids, and green tea which is a very rich source of flavan-3-ols. The review concentrates on human studies and where catabolites and related compounds were identified by mass spectrometric-based methods without recourse to the use of enzyme hydrolysis prior to analysis.

## 2. Coffee

**Figure 1:**
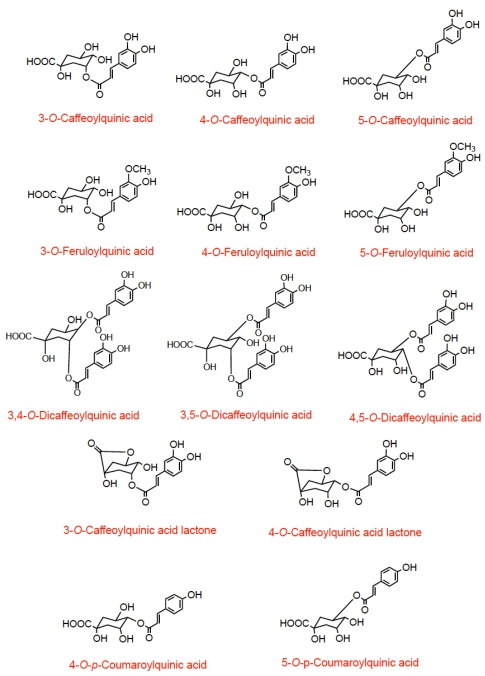
Structures of chlorogenic acids occurring in coffee.

Coffee, in different preparations, is widely consumed throughout the world, and contains high levels of phenolic compounds. A single serving provides between 20 and 675 mg of chlorogenic acids depending on the type of roast and the volume consumed and regular consumers an easily have an intake in excess of 1 g per day [10,11]. Chlorogenic acids are a group of compounds comprising hydroxycinnamates, such as caffeic acid, ferulic acid, and *p*-coumaric acid, linked to quinic acid to form a range of conjugated structures known as caffeoylquinic acids (CQA), feruloylquinic acids (FQA), and *p*-coumaroylquinic acids all of which exist in several isomeric forms [10]. As well as these compounds coffee also contains dicaffeoylquinic acids and caffeoylquinic acid lactones (CQAL) (Figure 1).

The literature describing the catabolism of coffee chlorogenic acids in human subjects is scarce and, in some instances, contradictory. A study by Nardini *et al.* [12] observed an increase of conjugated caffeic acid in plasma after the ingestion of 200 mL of coffee, while Rechner *et al.* [8] detected ferulic acid, isoferulic acid, dihydroferulic acid, 3-methoxy-4-hydroxybenzoic acid, hippuric acid and 3-hydroxyhippuric acid in urine from five human subjects after three ingestions of two cups of coffee at 4-h intervals. Monteiro *et al.* [13] reported the presence of unmetabolised CQAs in human plasma at a mean peak plasma concentration (*C_max_*) of 7.7 µmol/L 2.3 h (*T_ma_*_x_) after acute ingestion of coffee containing 3,395 µmol of CQAs. Despite the high *C_max_* of the CQAs, chlorogenic acids were not detected in urine collected 0–24 h after coffee intake. However, in a subsequent study by the same group, in which human volunteers consumed a coffee containing a much lower 451 µmol of chlorogenic acids, 4- and 5-*O*-CQAs were detected in sulfatase/glucuronidase-treated urine from some, but not all, subjects [14].

**Figure 2:**
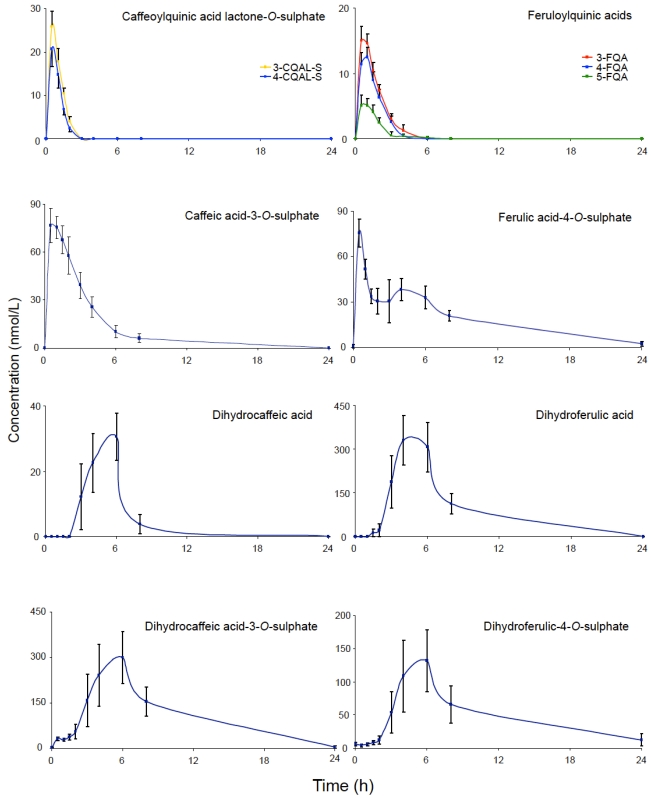
Plasma pharmacokinetic profiles of circulating chlorogenic acids and metabolites, following the ingestion of 200 mL of coffee by health human subjects.

The most recent and detailed research on the fate of chlorogenic acids after the ingestion of coffee is that of Stalmach *et al.* [15,16] who in studies with healthy humans and ileostomists, in which analysis comprised HPLC-MS^2^-based methodology, noted that during passage through the body extensive metabolism of chlorogenic acids occurs with some compounds being absorbed in the small intestine and others in the colon. The plasma pharmacokinetic profiles of circulating chlorogenic acids and their metabolites observed with healthy subjects with a functioning colon, after the ingestion of 412 µmol of chlorogenic acids are illustrated in Figure 2. Maximum *C_max_* values ranged from 6 nmol/L for 5-FQA to 385 nmol/L for dihydroferulic acid, with the duration for *T_ma_*_x_ extending from 0.6 h (ferulic acid-4-*O*-sulfate, 3-CQLA-*O*-sulfate) to 5.2 h (dihydroferulic acid). The compounds detected in highest concentrations in plasma were free and sulfated conjugates of dihydroferulic acid and dihydrocaffeic acid acid with *C_max_* values ranging from 41 to 385 nmol/L. The *T_max_* for these compounds was in a narrow range from 4.7 to 5.2 h, indicating absorption in the large intestine. Much shorter *T_max_* values of 0.6 to 1.0 h, indicative of small intestine absorption, were obtained with 5-*O*-CQA, two CQLA-*O*-sulfates and three FQAs, all of which had relatively low *C_max_* values (Figure 2). As noted by Stalmach *et al.* [15] most of the chlorogenic-derived compounds were rapidly removed from the circulatory system with elimination half-life (*T_1/2_*) values of 0.3 to 1.9 h. The only compounds with an extended *T_1/2 _* were dihydroferulic acid-4-*O*-sulfate (4.7 h), dihydroferulic acid-3-*O*-sulfate (3.1 h) and ferulic acid-4-*O*-sulfate which had an unusual biphasic plasma profile with dual *T_max_* values at 0.6 h and 4.3 h. The only unmetabolised compounds detected in plasma were three FQAs (Figure 2) and trace concentrations of 5-*O*-CQA (*C_max_*–2.2 nmol/L; *T_max_*–1.0 h). It is also of note that the free acid, dihydroferulic acid, as opposed to the more typical glucuronide and sulfate metabolites, was the principal component to accumulate in plasma which also contained dihydrocaffeic acid in a lower concentration (Figure 2).

The ileostomists drank a coffee with a very similar 385 µmol chlorogenic acid profile to that ingested by the health subjects [16]. Plasma was not investigated but analysis of the 0–24 h ileal fluid revealed the presence of 275 µmol of chlorogenic acids mainly, but not exclusively, as unmetabolised compounds. This indicates that *ca.* 30% of the chlorogenic acids were absorbed in the small intestine of the ileostomists and that in subjects with a functioning colon *ca.* 70% of intake will pass from the small to the large intestine.

The quantities of chlorogenic acids and their metabolites excreted in urine by healthy subjects and ileostomists over a 24 h period post-ingestion of coffee are summarised in Table 1. The healthy volunteers excreted a total of 120.2 µmol which corresponds to 29.2% of intake while urine from ileostomists contained 30.8 µmol which equates with only 8.0% of the ingested chlorogenic acids. This highlights the importance of the colon in the bioavailability of dietary chlorogenic acids.

**Table 1:** Urinary excretion of chlorogenic acid metabolites in 0–24 h urine of healthy subjects (n = 11) and ileostomists (n = 5) following the ingestion of 200 mL of coffee.

Chlorogenic acid and metabolites	Subjects without a colon (385 µmol ingested)	Subjects with a colon (412 µmol ingested)
3-*O*-Caffeoylquinic acid lactone-*O*-sulfate	0.6 ± 0.1	1.1 ± 0.1
4-*O*-Caffeoylquinic acid lactone-*O*-sulfate	0.4 ± 0.1	1.0 ± 0.1
3-*O*-Feruloylquinic acid	0.9 ± 0.2	1.2 ± 0.1
4-*O*-Feruloylquinic acid	0.9 ± 0.2	1.1 ± 0.1
5-*O*-Feruloylquinic acid	1.1 ± 0.2	1.0 ± 0.2
Ferulic acid-4-*O*-sulfate	9.9 ± 1.9	11.1 ± 1.6
Feruloylglycine	2.1 ± 0.3^a^	20.7 ± 3.9^b^
Dihydroferulic acid	n.d.^ a^	9.7 ± 2.0^b^
Dihydroferulic acid-4-*O*-sulfate	0.8 ± 0.2	12.4 ± 3.4
Dihydroferulic acid-4-*O*-glucuronide	n.d.	8.4 ± 1.9
Isoferulic acid-3-*O*-sulfate	0.2 ± 0.0	0.4 ± 0.1
Isoferulic acid-3-*O*-glucuronide	3.9 ± 0.8	4.8 ± 0.5
Dihydro-isoferulic acid-3-*O*-glucuronide	n.d.^a^	2.5 ± 0.4^b^
Caffeic acid-3-*O*-sulfate	6.2 ± 1.2	6.4 ± 0.8
Caffeic acid-4-*O*-sulfate	0.6 ± 0.1	0.6 ± 0.1
Dihydrocaffeic acid-3-*O*-sulfate	3.2 ± 0.9^a^	37.1 ± 8.2^b^
Dihydrocaffeic acid-3-*O*-glucuronide	n.d^a^	0.7 ± 0.2^b^
Total	30.8 ± 4.3 (8.0%)^a^	120.2 ± 17.0 (29.2%)

^a^ Data represent mean values in mmol ± SE. n.d. not detected. Different superscripts within rows indicate a statistical difference between the two sets of volunteers (Two-sample T-test, P-value < 0.05). Figures in bold italicised parentheses indicate excretion as a percentage of chlorogenic acid intake.

**Figure 3:**
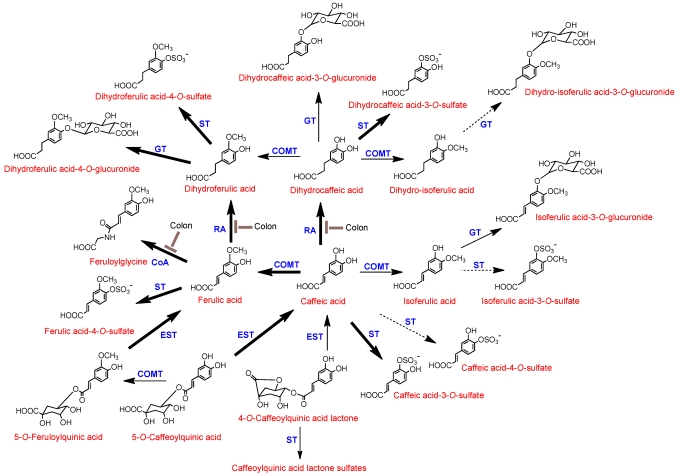
Proposed metabolism of chlorogenic acids following the ingestion of coffee by human volunteers. 5-*O*-CQA and 5-*O*-FQA are illustrated structures but their respective 3- and 4-isomers would be metabolized in a similar manner and likewise with 4-*O*-CQAL and 3-*O*-CQAL. COMT, catechol-*O*-methyltransferase; ET, esterase; RA, reductase; GT, UDP-glucuronyltransferase; ST, sulfuryltransferase; Co-A, co-enzyme A. Bold arrows indicate major routes, dotted arrows minor pathways. Steps blocked in subjects with an ileostomy and hence occurring in the colon are indicated.

The data presented in Table 1 show that absence of a colon had minimal impact of the excretion of CQAL-*O*-sulfates and FQAs, as well as caffeic, ferulic and isoferulic acid-*O*-sulfates. Furthermore, the data indicate that the small intestine is most probably the site for i) cleavage of quinic acid from CQAs and FQAs releasing caffeic acid and ferulic acid, (ii) metabolism of caffeic acid to its 3- and 4-*O*-sulfates, and ferulic acid to ferulic acid-4-*O*-sulfate and iii) the methylation of caffeic acid to form isoferulic acid and its subsequent 3-*O*-sulfation and glucuronidation. In contrast, there were major reductions in the excretion of dihydrocaffeic acid-3-*O*-sulfate, dihydrocaffeic acid-3-*O*-glucuronide, dihydroferulic acid and its glucuronide and sulfated derivatives, dihydro-isoferulic acid-3-*O*-glucuronide and feruloylglycine by the ileostomists. This demonstrates that the colon is the site for i) the conversion of ferulic acid to feruloylglycine and dihydroferulic acid and ii) metabolism of caffeic acid to dihydrocaffeic acid which is further metabolised to dihydro-isoferulic acid. Despite its dual plasma *T_ma_*_x_ at 0.6 and 4.3 h in healthy subjects (Figure 2), urinary excretion of ferulic acid-4-*O*-sulfate was unaffected by the absence of a colon (Table 1) indicating that its secondary plasma *T_ma_*_x_ is not a consequence of colonic absorption. Stalmach *et al.* [15,16] proposed the data obtained in their coffee feeding studies with healthy volunteers and ileostomists are in keeping with the metabolic routes illustrated in Figure 3. 

## 3. Green Tea

**Figure 4:**
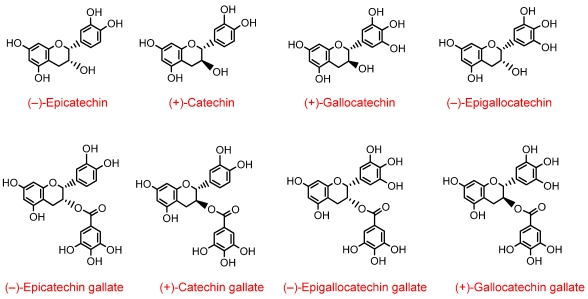
Structures of green tea flavan-3-ols.

Green tea produced by aqueous infusion of young leaves of *Camellia sinensis* is a rich source of several flavan-3-ols (Figure 4), typically, with (–)-epigallocatechin-3-*O*-gallate, (–)-epigallocatechin and (–)-epicatechin predominating [17]. Recently, detailed information on the bioavailability of these compounds has appeared in the literature. In one study, ten healthy human subjects consumed 500 mL of green tea containing 648 µmol of flavon-3-ols after which plasma and urine were collected over a 24 h period and analysed by HPLC-MS^2^ [3]. Plasma contained a total of twelve metabolites, in the form of *O*-methylated, sulfated and glucuronide conjugates of (epi)catechin and (epi)gallocatechin along with the native green tea flavan-3-ols (–)-epigallocatechin-3-*O*-gallate and (–)-epicatechin-3-*O*-gallate. Analysis of the pharmacokinetic profiles of these compounds, is presented in Table 2.

**Table 2:** Pharmacokinetic analysis of flavan-3-ols and their metabolites detected in plasma of healthy volunteers following the ingestion of 500 mL of green tea. Data expressed as mean values ± SE (n = 10).

Flavan-3-ols *(number of isomers)*	*C*_max _(nmol/L)	*T*_max _(h)
(Epi)gallocatechin-*O*-glucuronide *(1)*	126 ± 19	2.2 ± 0.2
4'-*O*-Methyl-(epi)gallocatechin-*O*-glucuronide *(1)*	46 ± 6.3	2.3 ± 0.3
4'-*O*-Methyl-(epi)gallocatechin-*O*-sulfates *(2)*	79 ± 12	2.2 ± 0.2
(Epi)catechin-*O*-glucuronide *(1)*	29 ± 4.7	1.7 ± 0.2
(Epi)catechin-*O*-sulfates *(2)*	89 ± 15	1.6 ± 0.2
*O*-Methyl-(epi)catechin-*O*-sulfates *(5)*	90 ± 15	1.7 ± 0.2
(–)-Epigallocatechin-3-*O*-gallate *(1)*	55 ± 12	1.9 ± 0.1
(–)-Epicatechin-3-*O*-gallate *(1)*	25 ± 3.0	1.6 ± 0.2

None of the flavan-3-ols were present in the circulatory system at 0 h but they were present in detectable quantities 30 min after green tea consumption. The main component to accumulate was an (epi)gallocatechin-*O*-glucuronide with *C_max_* of 126 nmol/L and a *T_max_* of 2.2 h while an (epi)catechin-*O*-glucuronide, probably the 3'-*O*-conjugate, attained a *C_max_* of 29 nmol/L with a 1.7 h *T_max_*. The unmetabolised flavan-3-ols (–)-epigallocatechin-3-*O*-gallate and (–)-epicatechin-3-*O*-galllate has *C_max_* values of 55 and 25 nmol/L after 1.6 and 2.3 h respectively. The *T_max_* durations ranged from 1.6 to 2.3 h (Table 2) and all the flavan-3-ols and their metabolites were present in only trace quantities after 8 h and were not detected in the 24 h plasma. This is indicative of absorption in the small intestine, a fact confirmed when a similar flavan-3-ol metabolite plasma profile was obtained after the ingestion of green tea by humans subjects with an ileostomy [5]. 

Urine collected 0–24 h after green tea consumption by healthy subjects with a functioning colon contained a similar spectrum metabolites of (epi)catechin and (epi)gallocatechin to plasma except for the appearance of two (epi)catechin-*O*-sulfates and an absence of unmetabolised flavan-3-ols (Table 3). The overall metabolite excretion was equivalent to 8.1% of the 648 µmol flavan-3-ol intake. However, there was notable distinction between the excretion of (epi)catechin and (epi)gallocatechin metabolites. The recovery of (epi)gallocatechin metabolites was 11.4% while that of (epi)catechin metabolites was 28.5% of the (–)-epicatechin and (+)-catechin intake (Table 3). These high levels of excretion are also in keeping with recoveries obtained in earlier studies with green tea, cocoa and related products [18,19,20] confirming that (–)-epicatechin and (+)-catechin, in particular, are highly bioavailable being absorbed and excreted to a much greater extent than other flavonoids with the possible exception of isoflavones [1,2,21].

**Table 3:** Quantification of the major groups of flavan-3-ol metabolites excreted in urine 0–24 h after the ingestion of 500 mL of Choladi green tea by ten human volunteers. Data expressed as mean values in µmol ± standard error (n = 10). Italicised figures in parentheses indicate amount excreted as a percentage of intake.

Flavan-3-ol metabolites *(number of isomers)*	0–24 h excretion (µmole)
(Epi)gallocatechin-*O*-glucuronide *(1)*	6.5
4'-*O*-Methyl-(epi)gallocatechin-*O*-glucuronide *(1)*	4.4
4'-*O*-Methyl-(epi)gallocatechin-*O*-sulfates *(2)*	19.8
(Epi)gallocatechin-*O*-sulfates *(3)*	2.6
**Total (epi)gallocatechin metabolites**	**33.3 *(11.4%)***
(Epi)catechin-*O*-glucuronide *(1)*	1.5 ± 0.3
(Epi)catechin-*O*-sulphates *(2)*	6.7 ± 0.7
*O*-Methyl-(epi)catechin-*O*-sulphates *(5)*	10.9 ± 1.2
**Total (epi)catechin metabolites**	**19.1 (*28.5%)***
**Total flavan-3-ol metabolites**	**52.4 *(8.1%)***

**Figure 5:**
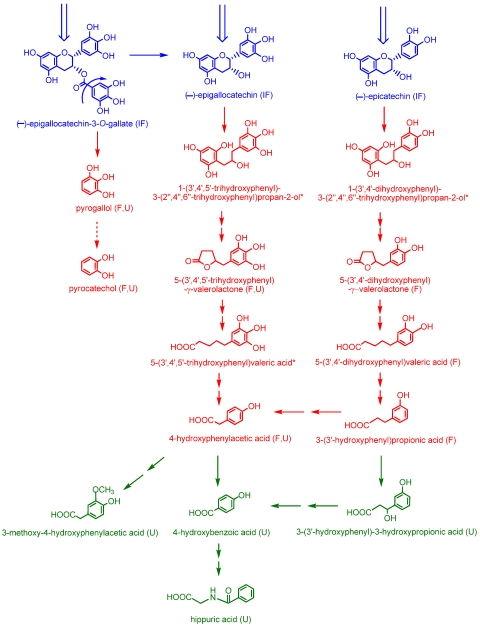
Proposed pathways involved in the colonic catabolism and urinary excretion of green tea flavan-3-ols. Following consumption of green tea more than 50% of the ingested (–)-epicatechin, (–)-epigallocatechin and (–)-epigallocatechin-3-*O*-gallate (blue structures) pass into the large intestine. When incubated with fecal slurries these compounds are catabolized by the colonic microflora probably via the pathways illustrated with red structures. Analysis of urine after green tea consumption indicates that some of the colonic catabolites enter the circulatory and undergo further metabolism before being excreted in urine. Green structures indicate catabolites detected in urine but not produced by fecal fermentation of (–)-epicatechin, (–)-epigallocatechin or (–)-epigallocatechin-3-*O*-gallate. The dotted arrow between pyrogallol and pyrocatechol indicate this is a minor conversion. Double arrows indicate conversions where the intermediate(s) did not accumulate and are unknown, although metabolism of 4-hydroxyphenylacetic acid to 3-methoxy-4-hydroxyphenylacetic acid probably proceeds via 3,4-dihydroxyphenylacetic acid. (IF) compounds detected in ileal fluid after green tea consumption; (F) catabolites detected in fecal slurries; (U) catabolites detected in urine; and (*) potential intermediates that did not accumulate in detectable quantities in either fecal slurries or urine.

Despite the relatively high absorption of flavan-3-ols in the small intestine, Stalmach *et al.* [5] report that after ileostomists drank green tea containing 634 µmol of flavan-3-ols, 69% of the intake as mixture of native compounds and metabolites was excreted in 0–24 h ileal fluid. Thus, in volunteers with a functioning colon most of the ingested flavan-3-ols will almost certainly pass from the small to the large intestine where their fate is a key part of the overall bioavailability equation. To mimic these events two sets of experiments were carried out. Firstly, 50 µmol of (–)-epicatechin, (–)-epigallocatechin and (–)-epigallocatechin-3-*O*-gallate were incubated under anaerobic conditions *in vitro* with fecal slurries and their degradation to phenolic acid by the bacterial microflora monitored. The main limitation of *in vitro* fermentation is that it may not fully depict the *in vivo* conditions. The removal of fecal material may alter the bacterial composition and thus may not represent the microflora in the colonic lumen and on the colonic mucosa where catabolism occurs *in vivo*. The accumulation and retention of the degradation products in the fermentation vessel makes collection, identification and quantification of the metabolites easier but is not necessarily representative of the events that occur *in vivo* where the actual concentration of a metabolite at any time interval is dependent on the combined rates of catabolism and absorption and this cannot be simulated *in vitro*. However, use of an *in vitro* model provides information on the types of breakdown products formed, helps elucidate the pathways involved, and the rate of catabolism can be determined. To complement the *in vitro* incubations, phenolic acids excreted in urine 0–24 h after i) the ingestion of green tea and water, in a cross over study, by healthy subjects, and ii) the consumption of green tea by ileostomist, was also investigated [22]. The data obtained in these studies provided the basis for the operation of the catabolic pathways that are illustrated in Figure 5. 

Some of these catabolites, such as 4-hydroxyphenylacetic acid and hippuric acid were detected in urine from subjects with an ileostomy indicating that they are produced in the body by additional routes unrelated to colonic degradation of flavan-3-ols. It is, for instance, well known that there are pathways to hippuric acid from compounds such as benzoic acid, quinic acids [23]*,* tryptophan, tyrosine and phenylalanine [24,25,26]*.* None-the-less, the elevated urinary excretion of hippuric acid and 4-hydroxyphenylacetic acid, occurring after green tea consumption, is likely to be partially derived from flavan-3-ol degradation. Earlier research showing statistically significant increases in urinary excretion of hippuric acid after consumption of both green and black tea by human subjects [23,27] supports this supposition.

Quantitative estimates of the extent of ring fission of the flavan-3-ol skeleton are difficult to assess because, as discussed above, the production of some of the phenolic acids was not exclusive to colonic degradation of flavan-3-ols. If these compounds, along with pyrogallol and pyrocatechol, which are derived from cleavage of the gallate moiety from (–)-epigallocatechin-3-*O*-gallate rather than ring fission, are excluded, excretion of the remaining urinary phenolic acids, namely 4-hydroxybenzoic acid, 3-methoxy-4-hydroxyphenylacetic acid, 3-(3'-hydroxyphenyl)-3-hydroxypropionic acid and 5-(3',4',5'-trihydroxyphenyl)-γ-valerolactone, was 210 µmol after ingestion green tea compared to 38 µmol after drinking water. The 172 µmol difference between these figures corresponds to a 39% degradation of the 439 µmol of flavan-3-ols entering the colon after consumption of green tea. Added to this is the *ca.* 8% excretion of glucuronide, sulfate and methylated flavan-3-ols originating from absorption in the small intestine. This combined estimate of a 47% recovery is nonetheless a minimum value because with the analytical methodology used some urinary catabolites will have escaped detection [22]. This will include glucuronide and sulfate metabolites of (–)-5-(3',4',5'-trihydroxyphenyl)-γ-valerolactone, (–)-5-(3',4' dihydroxyphenyl)-γ-valerolactone and (–)-5-(3',5' dihydroxyphenyl)-γ-valerolactone, which have been detected after green tea consumption with a cumulative 0–24 h excretion corresponding to 16% of flavan-3-ol intake [28,29,30]. More recently, in a similar study in which urine was collected for 24 h after green tea intake, valerolactone metabolites were excreted in quantities equivalent to 36% of intake [31]. When added to the 47% recovery estimated above, this gives a total excretion of 83% of intake. This figure is obviously an approximation because of factors such as different volunteers, flavan-3-ol intakes and analytical methodology. However, it does demonstrate that despite substantial modification as they pass through the body, there is a very high urinary recovery of flavan-3-ols, principally in the form of colon-derived catabolites.

## 4. Conclusions

The recent publications that are reviewed in this paper established that both flavan-3-ols in green tea and chlorogenic acids in coffee undergo extensive metabolism prior to absorption, initially in the small intestine prior to passage to the large intestine where the colonic microflora-mediated production of phenolic acids occurs. The chlorogenic acids and the flavan-3-ols produce their own unique spectrum of colonic catabolites which are excreted in urine in substantial amounts corresponding to 29% of intake after coffee consumption and *ca.* 83% after drinking green tea. Some, but far from all of the metabolites and colonic breakdown products appear transitorily in plasma, but seemingly are treated by the body as xenobiotics and are rapidly removed from the bloodstream. As a consequence, while analysis of plasma provides valuable information on the identity, *C_max_*  and *T_max_* values of circulating metabolites after acute supplementation, estimates of 'area under the curve' values do not provide accurate quantitative assessments of uptake from the gastrointestinal tract. Urinary excretion provides a more realistic figure but, as this does not include the possibility of metabolites being sequestered in body tissues, this too is an under estimate of absorption, but to what degree remains to be determined.

There is a growing realisation that the colon plays an important role in the bioavailability of dietary phenolic and polyphenolic compounds with the studies discussed in this review showing that even when absorption occurs in the small intestine, substantial quantities pass to the large intestine where the parent compounds and their catabolites can impact on both colonic health and the colonic microflora. The level of urinary excretion indicates that substantial quantities of the colonic catabolites are absorbed into the portal vein and pass through the body in the circulatory system prior to excretion. Some of these compounds may play a key role in the protective effects of a fruit and vegetable-rich diet as there is evidence that they have anti-inflammatory activity in experimental models [32].
